# Assessing parents’ self-efficacy to handle child obesity-related behaviors: validation of the Lifestyle Behavior Checklist in Iran

**DOI:** 10.1186/s41043-022-00288-9

**Published:** 2022-03-16

**Authors:** Nasrin Omidvar, Saba Narmcheshm, Hassan Eini-Zinab, Parisa Amiri, Sayyed Reza Sobhani, Azam Doustmohammadian

**Affiliations:** 1 Community Nutrition Department, Faculty of Nutrition Sciences and Food Technology, National Nutrition and Food Technology Research Institute, Shahid Beheshti University of Medical Sciences, No 46, West Hafezi St., Farahzadi Blvd., P.O. Box: 19395-4741, 1981619573 Tehran, Iran; 2 Research Center for Social Determinants of Health, Research Institute for Endocrine Sciences, Shahid Beheshti University of Medical Sciences, Tehran, Iran; 3 Gastrointestinal and Liver Diseases Research Center, Iran University of Medical Sciences, Tehran, Iran

**Keywords:** Childhood overweight, Obesity, Parenting, Self-efficacy, Scale validation, Lifestyle Behavior Checklist

## Abstract

**Background:**

The aim of the present study was to validate the Lifestyle Behavior Checklist (LBC) questionnaire, to measure Iranian parents' perceptions of their children's weight-related behaviors and their self-efficacy in dealing with those behaviors.

**Methods:**

The LBC was translated into Farsi. Face and content validity of the questionnaire was evaluated by an expert panel. A total of 213 mothers of 3–12-year-old children responded to the questionnaire. Criterion validity of the questionnaire was evaluated through comparing its result with a parenting style questionnaire. Principal component analysis (PCA) and confirmatory factor analyses (CFA) were used to evaluate construct validity of the questionnaire. Reproducibility was measured by twice administration of LBC, one month apart and using Spearman's rho correlation test. The questionnaire's internal consistency was assessed by calculating Cronbach's *α*.

**Results:**

LBC Problem scale was significantly correlated with authoritarian parenting style score, while the Confidence scale was significantly correlated with authoritative and negatively with permissive and authoritarian parenting styles. PCA suggested a six-factor construct, including, fussy eating, food-related problem behaviors, overeating behaviors, low interest in physical activity, poor self-image and sedentary behaviors. The results of CFA indicated acceptable fit indices for the proposed models. Both, Problem scale (Cronbach's *α* = 0.8) and Confidence scale (Cronbach's *α* = 0.95) had high internal consistency. Spearman correlation coefficients indicated acceptable reproducibility for both the Problem scale (*r* = 0.74) and the Confidence scale (*r* = 0.70).

**Conclusions:**

The Farsi version of LBC questionnaire is reliable and reasonably valid to measure Iranian mothers' perception of their children's weight-related problem behavior.

## Introduction

Childhood obesity is one of the major challenges of the twenty-first century [1] that can affect child development and accelerate adverse cardiovascular and metabolic risk factors [2], as well as psychosocial problems [3]. The prevalence of childhood overweight and obesity has increased at an alarming rate in almost all developed and several developing countries [4]. In addition, childhood obesity tracks into the adulthood [5], and its early onset can lead to higher morbidity and mortality risk in later years [6]. In Iran, as a middle-income country experiencing epidemiological and nutrition transition, the prevalence of overweight and obesity among school-age children is 7.9 and 5.6%, respectively [7].

In response to the childhood obesity epidemic, an increasing number of interventions have been designed aiming to prevent childhood excessive weight gain and reduce the risk of obesity [8]. Successful interventions have been indicated the pivotal role of parents to shape eating patterns and physical activity habits in their children which can strongly affect their weight status [9]. However, the majority of parents are shown to have problem handling their children obesity-related behaviors [10, 11]. Two levels of parenting-related behaviors are often distinguished, including parenting practices and general parenting styles [12]. Specific parenting practices are content-specific acts of parenting, such as rules about food intake or daily activities [13]. Parenting style captures two important elements of parental responsiveness and parental demandingness [14]. Both levels of parenting have shown to be of importance in describing and predicting children’s weight-related behaviors [12].

Self-efficacy is an important determinant of parenting behaviors that its impact has increasingly been emphasized [15]. Bandura defined self-efficacy as “a person’s belief in his/her capabilities to organize and execute the course of action required to manage prospective situations” [16]. However, the construct of weight-related parenting self-efficacy has been mostly neglected in the studies on child weight [17]. Over the past few years, many researchers have suggested low parental self-efficacy as a possible obstacle for parents ability to change their children’s nutrition and physical activity behaviors [17, 18]. Thus, it is noteworthy to identify initial parental challenges in managing children’s lifestyle behavior, as well as their self-efficacy. It should also be noted that parents of overweight and obese children compared to those with normal weight children may face additional difficulties. Obese and overweight children may have more physical health problems, poorer emotional functioning and increased school problems than their normal weight peers [19]. Therefore, measurement of parental confidence and skills regarding weight-related challenges are critical in child obesity prevention and management programs. In this regard, valid instruments that can capture specific characteristics of parents in dealing with children’s weight-related behaviors are required.

Considering the lack of a specific instrument to measure weight-related parental self-efficacy, West and Sanders developed and validated the Lifestyle Behavior Checklist (LBC). LBC measures parental perceptions of their children’s behavioral problems with overweight and obesity, as well as parents’ self-efficacy in dealing with these behaviors [20]. The tool was modified and applied in the Netherlands [21] and Sweden [22] and showed to be reliable and reasonably valid. In addition, the LBC scales are shown to be responsive to change following a parenting intervention [23].

The present study, as part of a larger pilot study on management of childhood obesity and overweight in Iran, aimed to evaluate the validity and reliability of the LBC questionnaire to measure Iranian parents’ perceptions of their children’s behavioral problems with overweight and obesity and their self-efficacy in dealing with these behaviors.

## Methods

This cross-sectional study was conducted from July to December 2013. The study was designed in two distinct phases: (1) translation and validation of the scale; and (2) confirmatory study to ensure the validity of the scale.

### Phase (1) Translation and validation of the Lifestyle Behavior Checklist

The 25-item Lifestyle Behavior Checklist is designed to assess parental perceptions regarding the extent of behavior problems of their overweight and obese children and parents’ confidence about managing their child problem behaviors. The checklist is composed of four sub-scales, including (1) misbehavior in relation to food (e.g., the child yells about food, eats unhealthy snacks, refuses to eat certain foods), (2) overeating (e.g., the child eats too much), (3, 4) emotional correlates of overweight (e.g., the child complains about being overweight) and physical activity (e.g., the child complains about being physically active) [20] and is consisted of two main parts, including (a) Problem scale and (b) Confidence scale. The Problem scale measures the extent to which parents perceive each of the 25 behaviors to be a problem behavior in their child, on a 7-point scale from 1 (not at all) to 7 (very much). The Confidence scale measures the extent to which parents feel confident about managing each behavior on a 10-point scale from 1 (certain I cannot do it) to 10 (certain I can do it).

The clinical cutoff values for the Problem scale are above 50 (range = 25–175) and for the Confidence scale under 204 (range = 25–250) [24].

*Translation*: The LBC was first translated into Farsi by four experts, including three nutritionists and a health education specialist. To produce a conceptual equivalence of translation to the original English questionnaire, all translators discussed any disparities and agreed on a single version. The final translated version was then translated back into English by a professional translator who was not involved in Farsi translation process and sent back to Dr. West via email. The meaning of the original LBC questions appeared similar in most cases.

*Face and content validity*: After finalizing the translation process, the questionnaire was pretested among 15 mothers who were part of the target population (but were not included in the final study) to ensure clarity in the linguistic and conceptual equivalence of the translations. The pretest was based on cognitive interviewing, i.e., using verbal probing techniques to identify the optimal format and wording of the questions. Only one item was changed during pretesting due to cultural acceptability (takes food from others instead of steals food from others). Subsequently, another meeting between the experts took place to finalize the questionnaire.

*Criterion validity*: Criterion validity of Farsi version of LBC was evaluated by comparing its result with a locally validated questionnaire on parenting styles. The latter questionnaire is the only valid questionnaire in Iran for assessing parenting styles [25]. The parenting style questionnaire has been developed for the purpose of measuring Baumrind's [27] permissive, authoritarian and authoritative parental authority prototypes [26]. It consists of 30 items and yields permissive, authoritarian and authoritative scores for both mother and father. Each of these parenting styles reflects different naturally occurring patterns of parental values, practices and behaviors [27]. Permissive parents were seen as more responsive than they are demanding. According to Baumrind’s perspective, permissive indulgent parents were “nontraditional and lenient, did not require mature behavior, allowed considerable self-regulation, and avoided confrontation.” Authoritarian parents, on the other hand, were seen as highly demanding and directive and not responsive. Authoritarian parents appear to provide well-ordered and structured environments with clearly stated rules. However, authoritative parents were both demanding and responsive. They are assertive, but not intrusive and restrictive [27].

Criterion validity was assessed using bivariate correlations (Spearman’s rho correlation tests) between the LBC scales and the parenting styles scores. The magnitude of the relationship (effect size, “*r*”) was used as a source of information. Interpretation of the strength of the effect size was based on Cohen’s descriptive guidelines. A correlation higher than or equal to 0.50 was regarded as large, between 0.30 and 0.50 as medium and those smaller than or equal to 0.20 as small effect size.

*Construct validity*: In the present study, principal component analysis (PCA) extraction was used to explore the existing factorial pattern. The number of factors was determined through evaluating four criteria: eigenvalues, percent of explained variance by each factor, scree plot and interpretability.

*Reliability*: Internal consistency of the scales was assessed using Cronbach’s alpha coefficient. To assess reproducibility, the LBC questionnaire was filled by 27 of the mothers twice, one month apart (test–retest).

*Participants*: The study sample consisted of 213 mothers who were selected through convenient sampling from those who were visiting primary healthcare centers in districts 4, 7 and 8 in the city of Tehran. Respondents were included if they were mothers of children aged 3–12 years and agreed to take part in the study by signing an informed consent.

*Data collection*: Mothers filled out the questionnaire in the waiting room of the selected health centers. Demographic information including mother and father’s age, education level, employment and marital status were obtained. Child characteristics (date of birth, gender, height and weight) were recorded out of the health records. Children’s BMI was calculated and recoded into BMI *Z*-scores curves. Weight status based on BMI Z-scores was classified into healthy weight (*Z*-score BMI for age between − 2 and + 1), overweight (between ≥ 1 and + 2) or obese (≥ + 2) as defined by WHO references in 2007 [28]. Children with underweight were excluded.

### Phase (2) Confirmatory study

In order to evaluate the factor structures identified through this analysis, mothers of 174 children aged 6–7 years were selected from districts 7 and 8 in Tehran city. To assess the consistency of the results, the selected samples were different from those studied in the previous phase. Written informed consent was obtained from students and their parents. Confirmatory factor analysis (CFA) was performed by AMOS 21.0, using the same parameters and fit indices, as phase 2.

*Discriminative validity*: To examine discriminative validity, group means for all the individual items of the Problem scale and the Confidence scale were compared between parents of children with normal weight and those with overweight or obese children (Table 3).

### Statistical analysis

Descriptive statistics were used to examine the normality and quality of the items. A quality item should have a mean that represents the middle of the response scale and a larger standard deviation. The total score of each scale was computed by summing up items related to the scale and used in the analysis. Data were presented as “Mean ± SD” for the quantitative continuous variables. Percentages of the categorical data were also calculated. All *p* values < 0.05 were regarded significant. Statistical Package for the Social Sciences (SPSS) for Windows, version 21.0; IBM Co., Armonk, NY, USA, was used for data analysis.

PCA was performed to determine the number and nature of underlying factors in the scale. Sampling adequacy was evaluated by Kaiser–Meyer–Olkin (KMO). Bartlett's test of sphericity and total variance explained were used for the evaluation of factor analysis. Factor loadings were used to keep or drop items. CFA was performed to test whether data fit the hypothesized measurement model that was extracted by PCA.

Weighted least squares (WLS) estimation method was used at CFA. Asymptomatic covariance matrix was considered as a weighted matrix. Goodness-of-fit indices (GFIs) and reasonable threshold levels of these indices for CFA were considered as *χ*^2^/*df* < 3, root mean square error of approximation (RMSEA) < 0.08, goodness-of-fit index (GFI) > 0.9 and adjusted goodness-of-fit index (AGFI) > 0/8 [29].

## Results

### Phase (1) Validation of the scale

A total of 213 mothers of 3–12-year-old children took part in the study. They were all the biological mothers of the children. Characteristics of the participants are summarized in Table 1. Of the children, 26.3% were overweight or obese.

**Table 1:** General characteristics of families participated in the study (*n* = 213)

Variable	Mean	SD
Mother age (year)	33.76	5.49
Father age (year)	39.03	5.62
Child age (year)	6.46	0.65

	Number	%
*Child’s sex*		
Girl	93	43.7
Boy	120	56.3
*Child weight status*		
Normal (> − 2 and < + 1)	157	73.7
Overweight (+ 1 to < + 2)	24	11.3
obese (+ 2 to < + 3)	18	8.5
Severely obese(≥ + 3)	14	6.6
*Mothers’ education*		
Below high school diploma	53	24.9
High school diploma	100	46.9
Associate's degree	14	6.6
Bachelor of science	34	16
Master of science	12	5.6
*Fathers’ education*		
Below high school diploma	67	31.6
High school diploma	74	34.9
Associate's degree	21	9.9
Bachelor of science	35	16.5
Master of science	15	7.1
*Mothers' job status*		
Housewife	176	82.6
Employed	37	17.4
*Fathers' job status*		
Employee	78	36.8
Self-employed	127	59.9
Other	7	3.3
*Family structure*		
Both parents	205	96.2
Only mother	8	3.8

*Criterion validity*: Problem scale score was positively correlated only with authoritarian style (*r* = 0.16, *p* = 0.014), while Confidence scale was positively correlated with authoritative parenting style (*r* = 0.14, *p* = 0.04) and negatively correlated with permissive (*r* =  − 0.14, *p* = 0.03) and authoritarian (*r* =  − 0.19, *p* = 0.004). In other words, mothers with authoritarian parenting style had a higher score on the Problem scale.

*Construct validity*: PCA on Problem scale resulted in extraction of 5 components. The KMO (Kaiser–Meyer–Olkin Measure of Sampling Adequacy) was 0.71 exceeding the recommended value of > 0.6, which indicates sufficient sample size. Bartlett’s test confirmed factor analysis appropriateness (*χ*^2^ = 1475.19, *df* = 300 and *p* < 0.001). The five-component solution accounted for 49% of the variance (Table 2). Item 17 in component 1 (fussy eating) and item 16 in component 2 (food-related problem behaviors) had low factor loading, and theoretically, they were meaningless; therefore, they were categorized as the sixth component. The six components were labeled as “fussy eating (FE),” including items 4, 5, 6 and 7, “food-related problem behaviors (FPB)” including items 3, 10, 11, 12, 13, 14 and 15, “overeating behaviors (OB)” including items 1, 2, 8 and 9, “low interest in physical activity (LPA)” including items 18, 19 and 20, “poor self-Image (PSI)” including items 21, 22, 23, 24 and 25 and “sedentary behaviors (SB)” including items 16 and 17.

**Table 2:** Factor loadings for rotated component matrix for responses to questions included in Problem scale LBC

Items	Fussy eating	Food-related problem behaviors	Overeating behaviors	Low interest in physical activity	Poor self-image	Extraction
4. Whinges or whines about food	**.812**	.001	− .113	.027	.007	0.672
6. Throws a tantrum about food	**.799**	.074	.016	.021	.143	0.665
7. Refuses to eat certain foods (i.e., fussy eating)	**.724**	− .011	− .108	.064	.115	0.554
5. Yells about food	**.610**	.051	.157	.001	.096	0.409
17. Spends too much time playing video or computer games	**.363**	.192	.197	.310	.040	0.305
13. Hides food	− .038	**.731**	.005	− .036	.096	0.547
11. Demands food when shopping or on outings	.155	**.603**	− .016	.077	.166	0.421
15. Eats food to comfort themselves when feeling let down or depressed	.036	**.596**	.348	.068	− .118	0.496
14. Steals food (e.g., from other children’s lunchboxes)	.000	**.580**	.101	.107	− .083	0.364
3. Eats unhealthy snacks	.399	**.446**	− .036	− .050	.069	0.367
12. Sneaks food when they know they are not supposed to	− .099	**.439**	.382	.108	.149	0.382
10. Requests food continuously between meals	.073	**.437**	.325	− .002	.235	0.357
16. Watches too much television	.324	.333	.102	.265	.185	0.331
2. Eats too much food	− .257	.101	**.726**	.004	− .018	0.604
9. Demands extra helpings at meals	− .048	.297	**.702**	.120	.055	0.601
1. Eats too quickly	.103	.054	**.679**	.096	− .110	0.495
8. Argues about food (e.g., when you say *No more*)	.284	.043	**.523**	.085	.169	0.392
19. Refuses to do physical activity	− .062	− .071	.116	**.848**	− .055	0.744
18. Complains about doing physical activity (e.g., *This is boring*, *I*’*m too tired*, *My leg hurts*)	.075	.100	.092	**.824**	.031	0.704
20. Complains about being unfit or feeling low in energy	.079	.129	.037	**.747**	.108	0.593
22. Complains about being teased	.053	.145	− .063	.104	**.811**	0.697
25. Complains about not fitting into clothes	.105	.122	− .022	− .027	**.617**	0.408
21. Complains about being overweight	.042	− .216	.448	.079	**.514**	0.519
23. Complains about not having enough friends	.096	.327	− .034	.197	**.454**	0.362
24. Complains about being unattractive	.245	− .088	.184	− .108	**.431**	0.299

Factor loadings within the accepted range are presented as bold

**Fig. 1:**
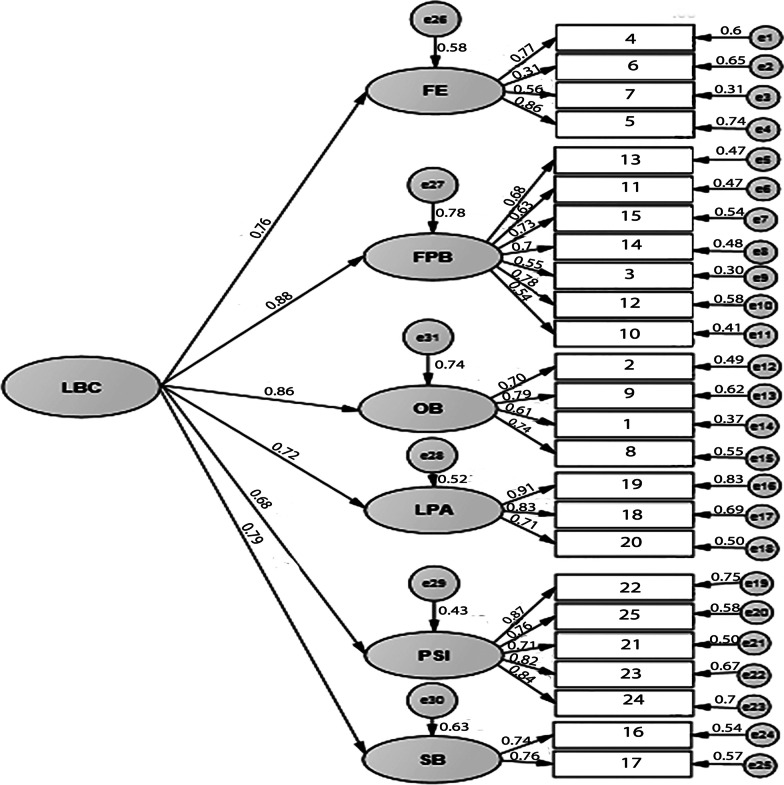
Confirmatory factor analysis of the Problem scale of the Lifestyle Behavior Checklist. The model shows acceptable fit to data (*χ*^2^/*df* = 1.699, *p* < 0.001; CFI = .847; GFI = .832, AGFA = .797, RMSEA = .064)

**Fig. 2:**
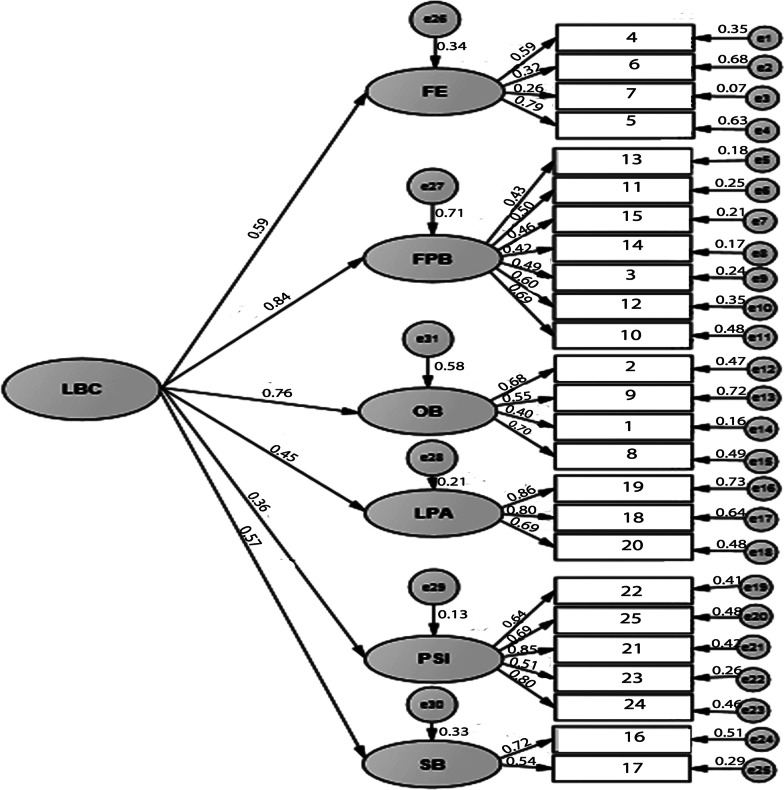
Confirmatory factor analysis of the Confidence scale of the Lifestyle Behavior Checklist. The model shows marginal acceptable fit to data (*χ*^2^/*df* = 2.074, *p* < 0.001; CFI = .880; GFI = .808, AGFA = .768, RMSEA = .079)

Second-order confirmatory factor analysis for Problem scale is shown in Fig. 1 (*χ*^2^/*df* = 1.699, *p* < 0.001; CFI = 0.847; GFI = 0.832, AGFA = 0.797, RMSEA = 0.064). All factor loadings were statistically significant (*p* < 0.001). Therefore, CFA confirmed the accuracy of the 6 component structure. Since both parts of the questionnaire (Problem scale and Confidence scale) are designed to estimate a number of common concepts, the same model of six components was used for Confidence scale and confirmed by CFA (*χ*^2^/*df* = 2.074, *p* < 0.001; CFI = 0.880; GFI = 0.808, AGFA = 0.768, RMSEA = 0.079) (Fig. 2).

*Discriminant validity*: To examine discriminant validity, group means for all items of the Problem scale and the Confidence scale were provided and compared between parents of children with normal weight and those whose children were overweight or obese (Table 3). On the Problem scale, 9 of the 25 items significantly differed between the groups. The mean and standard deviation of total scores on the Problem scale for parents of children with normal weight were lower (58.2 ± 15.2) than those for parents of overweight or obese children (62.8 ± 19.9); however, they were not significant.

**Table 3:** Group means for the LBC items based on child weight status (*n* = 213)

	Problem scale				Confidence scale			
	Healthy weight (*n* = 157)	Overweigh/obese (*n* = 56)	*p*-value	*t*	Healthy weight (*n* = 157)	Overweight/obese (*n* = 56)	*p*-value	*t*
1. Eats too quickly	1.77 ± 1.22	2.80 ± 1.63	< 0.001*	− 4.40	6.65 ± 2.47	6.88 ± 2.44	0.55	− 0.65
2. Eats too much food	2.04 ± 1.48	3.16 ± 1.61	< 0.001*	− 5.01	6.49 ± 2.67	6.52 ± 2.58	0.97	− 0.11
3. Eats unhealthy snacks	2.71 ± 1.63	2.64 ± 1.56	0.93	− 0.07	6.93 ± 2.65	7.45 ± 2.61	0.18	− 1.26
4. Whines about food	3.19 ± 1.80	2.73 ± 1.92	0.044*	1.64	6.74 ± 2.46	6.89 ± 2.61	0.52	− 0.47
5. Yells about food	1.99 ± 1.45	1.67 ± 1.29	0.15	1.27	7.51 ± 2.30	7.66 ± 2.44	0.43	− 0.57
6. Throws a tantrum about food	2.48 ± 1.63	1.89 ± 1.88	0.003*	2.20	6.98 ± 2.47	7.32 ± 2.67	0.19	− 0.97
7. Refuses to eat certain foods (i.e., fussy eating)	3.12 ± 1.86	2.53 ± 1.63	0.005*	2.39	6.51 ± 2.60	6.77 ± 2.89	0.41	− 0.59
8. Argues about food (e.g., when you say *No more*)	1.91 ± 1.34	2.75 ± 1.86	< 0.001*	− 3.20	7.05 ± 2.61	6.23 ± 2.77	0.054	1.88
9. Demands extra helpings at meals	2.01 ± 1.54	2.91 ± 1.69	< 0.001*	− 3.58	7.24 ± 2.62	6.88 ± 2.69	0.30	0.85
10. Requests food continuously between meals	3.57 ± 1.87	3.60 ± 1.89	0.83	0.18	6.47 ± 2.55	6.11 ± 2.93	0.55	0.82
11. Demands food when shopping or on outings	4.03 ± 1.82	3.85 ± 1.61	0.57	0.64	6.98 ± 2.53	6.98 ± 2.49	0.97	0.01
12. Sneaks food when they know they are not supposed to	1.60 ± 1.19	1.78 ± 1.21	0.12	− 1.13	7.61 ± 2.49	7.07 ± 2.67	0.2	1.29
13. Hides food	1.40 ± 1.11	1.25 ± 0.87	0.45	1.03	7.80 ± 2.75	7.57 ± 2.84	0.62	0.43
14. Takes food (e.g., from other children’s lunchboxes)	1.21 ± 0.66	1.38 ± 1.06	0.58	− 1.09	8.34 ± 2.26	7.86 ± 2.82	0.63	0.96
15. Eats food to comfort themselves when feeling let down or depressed	1.48 ± 1.11	1.60 ± 1.63	0.08	− 0.92	7.57 ± 2.69	7.29 ± 2.51	0.33	0.57
16. Watches too much television	4.21 ± 2.04	4.09 ± 1.98	0.75	0.28	6.25 ± 2.66	6.27 ± 2.69	0.93	− 0.07
17. Spends too much time playing video or computer games	2.94 ± 2.11	2.96 ± 2.13	0.86	− 0.06	6.75 ± 2.66	6.75 ± 2.80	0.68	− 0.30
18. Complains about doing physical activity	2.49 ± 1.73	2.98 ± 2.02	0.2	− 1.63	6.96 ± 2.25	7.00 ± 2.16	0.94	− 0.16
19. Refuses to do physical activity	1.74 ± 1.26	2.29 ± 1.57	0.005*	− 2.43	7.29 ± 2.42	6.86 ± 2.65	0.32	1.04
20. Complains about being unfit or feeling low in energy	2.33 ± 1.56	2.65 ± 1.71	0.22	− 1.07	7.26 ± 2.35	6.64 ± 2.46	0.1	1.65
21. Complains about being overweight	1.13 ± 0.66	2.13 ± 1.90	< 0.001*	− 4.00	7.82 ± 2.64	7.13 ± 2.61	0.057	1.37
22. Complains about being teased	2.97 ± 2.21	3.11 ± 2.11	0.59	− 0.30	7.90 ± 2.13	7.14 ± 2.58	0.08	1.86
23. Complains about not having enough friends	2.51 ± 2.01	2.73 ± 1.99	0.22	− 0.85	7.63 ± 2.40	7.63 ± 2.20	0.76	− 0.08
24. Complains about being unattractive	1.32 ± 0.87	1.18 ± 0.61	0.47	1.14	8.09 ± 2.45	7.73 ± 2.98	0.68	0.68
25. Complains about not fitting into clothes	2.09 ± 1.73	1.98 ± 1.67	0.75	0.10	8.07 ± 2.17	7.36 ± 2.81	0.17	1.53
Total Problem scale	59.24 ± 15.22	62.85 ± 19.9	0.2	− 1.57				
Total Confidence scale					179.92 ± 43.27	175.96 ± 48.66	0.68	0.56

*Statistically significant

On the Confidence scale, no significant difference was observed between the two group’s total scores. However, parents of overweight or obese children scored lower on 14 of the items and two of them were close to statistically significant: argues about food (e.g., when you say no more) (*p* = 0.054) and complains about being overweight (*p* = 0.057).

*Reliability*: Both Problem scale (Cronbach’s *α* = 0.80) and Confidence scale (Cronbach’s *α* = 0.95) had high internal consistency. Internal consistency of Problem scale components (sub-scales) included: fussy eating 0.78, food-related problem behaviors 0.67, overeating behaviors 0.68, low interest in physical activity 0.76, poor self-image 0.58 and sedentary behaviors was 0.61. Internal consistency of Confidence scale components was as follows: fussy eating 0.82, food-related problem behaviors 0.85, overeating behaviors 0.8, low interest in physical activity 0.85, poor self-image 0.89 and sedentary behaviors was 0.71. Poor self-image and sedentary behaviors scales had the lowest Cronbach’s *α*.

Means and standard deviations of the Problem scale and Confidence scale are presented in Table 3. Based on Pearson correlation coefficient, both Problem scale (*r* = 0.606, *p* = 0.001) and Confidence scale (*r* = 0.751, *p* < 0.001) had acceptable test–retest reliability. Spearman correlation between Problem scale and Confidence scale showed a significant negative correlation that means high scores on the Problem scale correlated with lower scores on the Confidence scale (*r* =  − 0.38, *p* < 0.0001). There was no significant difference in the Problem scale and Confidence scale of mothers according to the sex of their child.

The maximum score in the Confidence scale was related to item 14 (takes food) in both normal and overweight/obese children and the minimum score in overweight/obese children was related to item 10 (Requests food continuously between meals).

## Discussion

This study showed that LBC is a relatively valid and reliable tool to assess children’s obesity-related behaviors and parental self-efficacy to handle these behaviors in the Iranian population. In the present study, evaluating the construct of the questionnaire resulted in 6 components (fussy eating, food-related problem behaviors, overeating behaviors, low interest in physical activity, poor self-Image and sedentary behaviors). Previous studies on the psychometric analysis of LBC have all used EFA [20, 22], except for Gerards et al. study in the Netherlands [21]. The study by West et al. for Problem scale EFA resulted in 4 factors [30] (misbehavior in relation to food, overeating, emotional correlates of being overweight and physical activity); however, CFA was not done. On the other hand, in Ek study, 5 components were extracted, including Overeating, Physical Activity, Emotional correlates of being overweight, Misbehavior in relation to food and Screen Time. In the original model, sedentary behaviors were part of the physical activity scale, but based on Ek study and the present study, they were identified as two separate dimensions. The three dimensions that are present in all 3 studies include overeating, physical activity and emotional correlates of being overweight. In general, the six components scale adopted in the current study is closer to the modified scale by Ek; however, the correlations coefficients are smaller. Ek et al. omitted 6 items [3, 4, 7, 13, 20, 23] in their final model [22]. In addition, poor fit to the target population, as well as cultural and age differences between the study subjects may have had an effect on these results.

In estimating dimensions of the model, due to the lack of consensus among Structural Equation Model (SEM) specialists, several model fit indices have been used, including Chi-square (*χ*^2^), goodness-of-fit index (GFI), the adjusted goodness-of-fit index (AGFI) and root mean square error of approximation (RMSEA) and Comparative Fit Index (CFI) [31]. The RMSEA is currently the most popular measure of fitness which was almost within the acceptable range (≤ 0.08) in both parts of LBC (0.06 and 0.07 in Problem scale and Confidence scale, respectively). Based on the results of SEM, the optimum model of the scale is marginally acceptable.

Discriminate validity of the Problem scale showed a significant difference in 8 items, mostly related to overeating and fussy eating. There was no significant difference in Confidence scale scores between mothers of normal weight and overweight/obese children that indicated its poor discriminatory function. Overall, the Confidence scale seemed to be less sensitive than Problem scale.

In the present study, compared to Ek et al. and Gerards et al. reports, the mean score of mothers of overweight/obese children was higher in the Problem scale and lower in the Confidence scale. It can be concluded that in the present study mothers have more problems with child obesity-related behaviors while having lower self-efficacy to deal with the problems.

West et al. reported statistically significant differences between groups with different child weight status, for both scales [20]. However, they did not consider overweight children, when comparing parents of healthy weights with those of obese children. This difference in the samples may be responsible for higher mean scores of parents of Australian obese children on all Problem scale items compared to those of the parents of overweight and obese children in our study. In the present study, compared with Ek [22] and West’s study, each of the two parts of LBC had less discriminatory function. However, the internal consistency of the scales was similar to what was reported by West and Gerards [21].

This is the first study evaluating the psychometric properties of the LBC in Iran. Considering a heterogeneous sample of children with weight status, as well as a high response rate (75%) are strengths of the present study. However, the present study had some limitations that should be considered when evaluating its results. First, the weight and height measures of children were self-reported. Lack of measurement of mother’s weight is another limitation of this study. Also, to achieve more accurate results, it would be better to use another criterion measure in addition to Baumrind questionnaire. The cross-sectional design of the present study does not allow any conclusion on the causal effect of child behavior, parenting styles and self-efficacy. Considering different dimensions of validity parameters, more studies on Iranian mothers are recommended.

## Data Availability

It will be available from the author upon reasonable request.
